# Wearing a Cooling Vest During Half-Time Improves Intermittent Exercise in the Heat

**DOI:** 10.3389/fphys.2019.00711

**Published:** 2019-06-07

**Authors:** Yudai Chaen, Sumire Onitsuka, Hiroshi Hasegawa

**Affiliations:** ^1^Graduate School of Integrated Arts and Sciences, Hiroshima University, Higashihiroshima, Japan; ^2^Faculty of Human Health Science, Hiroshima Bunka Gakuen University, Kure, Japan

**Keywords:** intermittent exercise, cooling vest, skin temperature, heat, thermal comfort

## Abstract

Endurance and intermittent exercise performance are impaired by high ambient temperatures. Various countermeasures are considered to prevent the decline in exercise performance in the heat, convenient, and practical cooling strategies attracts attention. The purpose of this study was to investigate the effect of wearing a new type of cooling vest which cooled torso and neck during half-time (HT) on intermittent exercise performance that imitated intermittent athletic games. All measurements on the experiments were carried out with the bicycle ergometer. Eight male soccer players performed a familiarization session and two experimental trials of a 2 × 30 min intermittent cycling exercise protocol, which consisted of a 5 s maximal power pedaling (body weight ×0.075 kp) every minutes separated by 25 s unloaded pedaling (80 rpm) and rest (30 s) in the heat (33.0°C; 50% relative humidity). The two trials included cooling-vest condition (VEST) and control condition (CON), and the difference is with or without wearing cooling vest imposed for 15 min at HT. Mean and peak power output, rectal (Tre) and skin temperature (neck, upper back, chest, right upper arm, and thigh), heart rate (HR), deep thigh temperature, rating of perceived exertion (RPE), and thermal comfort (TC) and thermal sensation (TS) were measured. Mean power output at 2nd half was significantly greater (*p* < 0.05) in VEST (3rd trial: 589 ± 58 W, 4th trial: 584 ± 58 W) than in CON (3rd trial: 561 ± 53 W, 4th trial: 561 ± 53 W). HR were significantly lower in VEST during HT and higher in VEST at the last maximal pedaling (*p* < 0.05). At the end of HT, neck skin temperature and mean skin temperature were significantly lower in VEST (32.04 ± 1.47°C, 33.76 ± 1.08°C, respectively) than in CON (36.69 ± 0.78°C, 36.14 ± 0.67°C, respectively) (*p* < 0.05). During 2nd half, TS, TC, and RPE were significantly lower in VEST than in CON (*p* < 0.05). There was no significant difference in Tre and deep thigh temperature throughout each conditions. These results indicate that wearing a new type of cooling vest during HT significantly improves intermittent exercise performance in the heat with decreased neck and mean skin temperature and improved subjective responses.

## Introduction

In many team sports, athletes frequently perform in the heat, and are required to sustain exercise performance. Compared to the temperate environment, the core and skin temperatures increase in the heat, resulting in increased cardiovascular and metabolic strain and thermal perceptual load, as well as decreased exercise performance. Given that Tokyo 2020 Olympic and Paralympic games will be held in extremely hot and humid conditions (=33°C, 70–80% relative humidity), athletes should begin to prepare for this. Therefore, various countermeasures are considered to prevent the decline in exercise performance in the heat, requiring convenient and practical cooling strategies. Although it is well established that cooling interventions, such as water immersion or cooling with a large fan, are effective in normalizing the body temperature, these methods are not practical in the actual sports field (Quod et al., 2006). Additionally, the use of cooling vests is popular in many field team-sports due to their practicality and ease of use. Thus, wearing a cooling vest, which can be used conveniently, is one of the methods to prevent the decline in exercise performance in the heat (Randall et al., 2015).

However, details of the mechanism underlying the decline in exercise performance in the heat have not been clarified yet, but it is known that the central nervous system is involved (Nybo and Nielsen, 2001). Signals sensed from the peripheral thermoreceptors, such as in the skin, are sent to the hypothalamus, which is the center of temperature regulation (Tucker and Noakes, 2009). It has been suggested that the hypothalamus that senses high skin temperature or high thermal perception selectively regulates exercise intensity to complete an exercise task within a range not exceeding the critical limiting temperature. Therefore, wearing a cooling vest has a beneficial effect in reducing skin temperature and thermal perception and in relieving thermal strain sent from the peripheral thermoreceptors to the hypothalamus; thus, the selective decrease in exercise intensity is suppressed and endurance exercise performance is improved (Faulkner et al., 2015; Schmit et al., 2017). Previous studies that investigated improvement in exercise performance by wearing a cooling vest adopted an endurance exercise protocol such as self-paced trials (Arngrimsson et al., 2004; Faulkner et al., 2015; Schmit et al., 2017). However, intermittent sporting activities, such as soccer, rugby, and basketball, are also impaired when the ambient temperature is elevated (Duffield and Marino, 2007; Castle et al., 2006). As with endurance exercise, intermittent performance deteriorates in the heat (Drust et al., 2005). Furthermore, it has been suggested that intermittent exercise in the heat increases the thermal and metabolic strain further compared to endurance exercise (Ekblom et al., 1971; Mora-Rodriguez et al., 2008). Success in intermittent sports is greatly linked to the ability to perform repeated bouts of high intensity sprint exercises (Tyler et al., 2015). In a previous study, the potential cause of the decline in sprint performance observed in team sports activities in the heat was considered to be a decline in the function of the central nervous system caused by an increase in core, cerebral, and skin temperatures (Girard et al., 2015). Therefore, the physiological mechanism of declining intermittent exercise performance in the heat is similar to that of declining endurance exercise performance, and the selective decrease in exercise intensity by the motor cortex via thermal information from hypothalamus may also be caused by intermittent exercise performance. Therefore, wearing a cooling vest can suppress the selective decrease in exercise intensity and improve intermittent exercise in the heat.

Intermittent athletic games, such as soccer and rugby, have short rest periods between the exercise bouts and some cooling interventions are possible (Arngrimsson et al., 2004); countermeasures of heat are required for practical and convenient cooling interventions. In previous studies of pre-cooling methods, lower body cooling using ice packs improved subsequent sprint performance (Castle et al., 2006). However, it is also suggested that sufficient re-warming is required after cooling, since high-intensity exercise performance declined with decreasing the temperature of working muscles (Sleivert et al., 2001). It is difficult to do sufficiently re-warming in a short time such as during a half-time (HT); thus, it may be inappropriate to cool the working muscles directly during HT. Therefore, it is considered that wearing a cooling vest, which decreases skin temperatures without decreasing working muscle temperatures, can be effective because it can continue cooling even during HT. The purpose of this study was to investigate the effect of wearing a cooling vest, which can cool the torso and neck regions during HT, on intermittent exercise performance, imitating intermittent athletic games. We hypothesized that wearing a cooling vest which cooled the torso and neck regions decreases skin temperature and improves subjective sensation, and subsequent intermittent exercise performance is improved in the heat.

## Materials and Methods

### Participants

Eight non-heat-acclimated male soccer players (age: 21 ± 1.6 years, height: 174 ± 5 cm, mass: 64 ± 4 kg) volunteered for this study. They abstained from alcohol and caffeine consumption 24 h before the experiment, and they ingested same food and drink (Calorie Mate and Energen; Otsuka Pharmaceutical Co., Ltd., Japan) 2 h before the testing. All trials took place during the winter season with mean temperatures ranging from 10°C to 19°C to avoid the influence of natural heat acclimatization. The study procedures were approved by the Ethics in Human Research Committee of Hiroshima University, and all participants signed an informed consent form before the start of the study.

### Experimental Design

Participants completed one familiarization session before completing two experimental sessions in a randomized cross-over design. In one experimental session, participants wore the cooling vest (VEST) at the HT, and for the other session, they wore the cooling vest without ice packs (CON) at the HT. In the familiarization trial, they performed with the same measurement and protocol as the CON. Each session was separated by at least 4 days and completed at the same time of day to control for the effect of circadian rhythm on body temperature. All sessions took place in a climate environmental chamber in hot ambient conditions (33.0°C, 50% relative humidity). During all sessions, participants were allowed to consume water (33°C) up to 750 ml during the 2-min rest and HT, and all participants drank all the water.

### Exercise Protocol

**FIGURE 1:**
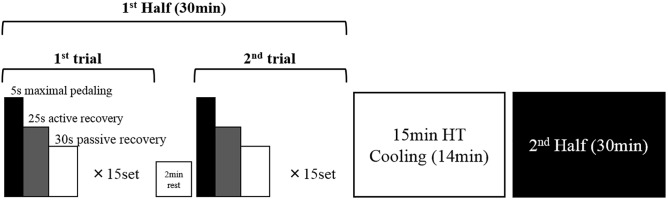
Exercise protocol, HT: half time.

Given that the measuring equipment was attached in all participants in the heat, they stayed in the heat for 30 min before the start of the exercise. All sessions were completed with the use of a cycling ergometer (POWERMAX-V2; COMBI, Japan). Participants completed warm-up exercises consisting of 7 min of cycling (body mass × 0.01 kp) and 3 sets of maximal pedaling (body mass × 0.075 kp) at 2 min before the exercise protocol. Participants completed a laboratory-based intermittent exercise protocol designed to replicate the demands of actual intermittent athletic games. The protocol consisted of two 30-min halves separated by a 15-min HT, with the half consisting of two trials separated by a 2-min rest period. One set consisted of a 5-s maximal pedaling (body mass × 0.075 kp), 25-s active recovery (no load, 80 rpm), and 30-s passive recovery. One trial consisted of 15 sets. Participants performed a total of four trials (Figure 1).

### Cooling Intervention

The cooling vest (Mizuno Co., Ltd., Japan), which can cool the neck, upper body, back, and side, was used during HT. Immediately after the 2nd trial ended, participants took off their underwear and training shirt, wiped their sweat, changed into a new training shirt, and wore the cooling vest. Given 1 min was required to change clothes, the actual cooling time was 14 min. In the VEST, ice packs (approximately -1°C) of gel material frozen in the freezer beforehand were put inside the pockets of the cooling vest. Since it was kept in the freezer until immediately before cooling, the cooling effect remained for the duration of 14 min. On the other hand, in the CON, cooling vests with similar weight as the used in the VEST (1.9 kg) were used.

## Measurements

### Performance Index

We measured the mean and maximal power outputs as the intermittent exercise performance. The mean power output was an average of 5-s maximal pedaling, and the maximal power output was calculated by the following equation:

Maximal power output=body mass × 0.075 kp × maximum rotation speed.

### Physiological Index

In this study, rectal (Tre), skin, and deep thigh temperatures, heart rate (HR), blood lactate, dehydration rate, and urine specific gravity were measured. Participants self-inserted a rectal probe approximately 10 cm past the anal sphincter. Skin temperatures were measured by attaching a thermistor probe on the neck, upper back, chest, upper arm, and thigh with active flex (BAND-AID; Johnson & Johnson, Japan) and insulation material seal (temperature insulation pad; Nihon Kohden Corporation, Japan) to accurately measure skin temperature. The mean skin temperature (Tsk) was calculated using the formula developed by Roberts et al. (1977):

$$T_{\mathrm{sk}}=(0.43\;\times \;T_{\mathrm{chest}})+(0.25\;\times \;T_{\mathrm{upper}\;\mathrm{arm}})+(0.32\;\times \;T_{\mathrm{thigh}}).$$

Participants also wore a HR monitor (model RS400; Polar Electro Oy, Kemple, Finland) that was attached before entering the environmental chamber. Deep thigh temperature measured by the deep body temperature monitor (CM-210, Terumo Co., Ltd., Japan) which detects the tissue temperature 5–10 mm below the skin surface using the zero heat flow method (Yamakage and Namiki, 2003). This monitor measures skin surface temperature beneath a thermal insulating pad containing a heater, which equilibrates the skin temperature with the deep tissue temperature when heat flow from the skin is maintained at zero. The consistency between muscle temperature measured using a needle thermocouple and the zero heat flow method was evaluated previously (Togawa et al., 1976; Wakabayashi et al., 2018). Tre, skin, and deep thigh temperature, HR were measured every 3 min during the experiment. Blood lactate was taken from the fingertip before starting the exercise and at the end of the trials and analyzed using an automated blood lactate analyzer (Lactate Pro; Arkrey, Japan). The body weight was measured using a scale (UC-300, A&D Co., Ltd., Japan) with the participants naked. The urine specific gravity was measured using a digital urine specific gravity refractometer (UG-D; Atago Co., Ltd., Japan) before and after the experiment. Changes in nude body mass were used to estimate gross sweat loss adjusted for fluid intake.

### Perceptual Index

Upper body thermal sensation and thermal comfort (TSupper, TCupper), neck thermal sensation and thermal comfort (TSneck, TCneck), and whole body thermal sensation and thermal comfort (TS, TC) were taken every 3 min during the experiment. TS and TC were rated using a 13-point scale that ranged from -6 (very cold) to 6 (very hot) and -6 (very uncomfortable) to 6 (very comfortable) (Olesen and Brager, 2004). Rating of perceived exertion (RPE) (Borg, 1973) was taken every 3 sprints in all trials.

### Statistical Analyses

All statistical calculations were performed using SPSS version 25.0. Mean power output, maximal power output, Tre, HR, Tsk, all skin temperatures, deep thigh temperature, blood lactate, all TS and TC, and RPE were analyzed using two-way repeated measures ANOVA (conditions × time). Dehydration rate and urine specific gravity were analyzed using one-way ANOVA (conditions). Where significant effects were identified, *post hoc* pairwise comparisons with Bonferroni correction were conducted. Where an interaction effect was observed, a paired samples *t*-test was conducted with Bonferroni correction applied. All data were checked for normal distribution by Kolmogorov–Smirnov test, and the violation of sphericity prior to analysis and Greenhouse-Geisser epsilon correction were used to adjust the degrees of freedom. The accepted level of significance for all analyses was *p* < 0.05. All data were presented as mean ± *SD*.

## Results

### Performance

**FIGURE 2:**
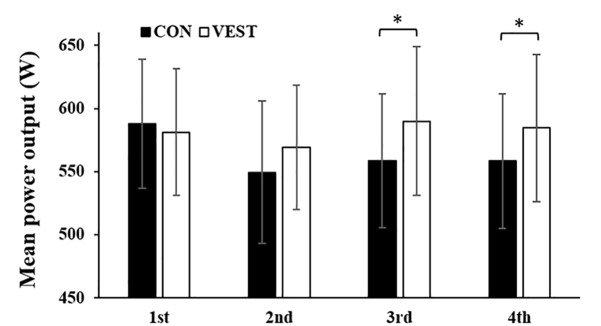
Power output per trial with and without wearing a cooling vest during half time. ^∗^: significantly different between the two conditions. The values are shown as mean ± *SD*. *p* < 0.05.

Mean power output in the 2nd half was significantly greater (*p* < 0.05; Figure 2) in VEST (3rd trial: 589 ± 58 W, 4th trial: 584 ± 58 W) than in CON (3rd trial: 561 ± 53 W, 4th trial: 561 ± 53 W). Seven out of the eight participants had improved mean power output in the 3rd trial in VEST than in CON, and all eight participants had improved mean power output in the 4th trial in VEST than in CON. Maximal power output was not significantly different between the two conditions.

### Body Temperature

**FIGURE 3:**
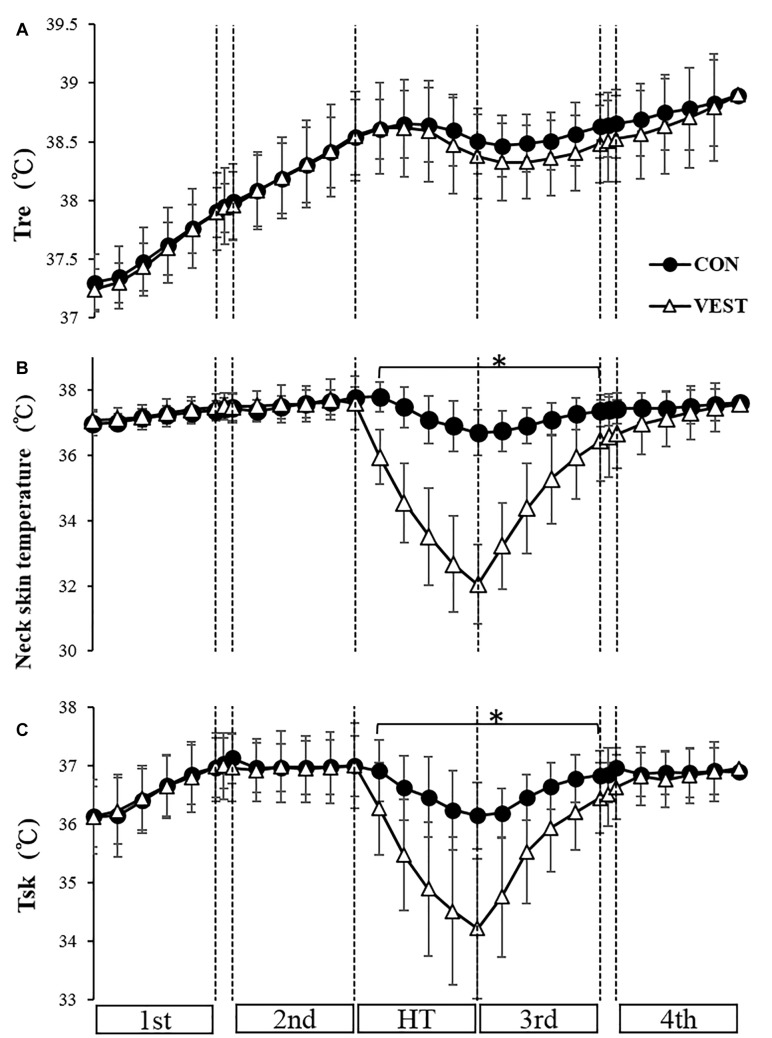
Change in rectal temperature **(A)**, neck skin temperature **(B)** and mean skin temperature **(C)** during exercise protocol. ^∗^: significantly different between the two conditions. The values are shown as mean ± *SD. p* < 0.05.

Baseline values of all body temperatures were taken at the start of the 1st trial. Tre at the end of HT was 0.13°C lower in VEST (38.3 ± 0.4°C) than in CON (38.5 ± 0.3°C); however, there were no significant differences between the two conditions (Figure 3A). Neck skin temperature and Tsk from the start of HT to the end of the 3rd trial was significantly lower in VEST than in CON (*p* < 0.05; Figure 3B,C). Owing to the failure of the measuring equipment, upper back skin temperature was analyzed in 6 participants. Chest and upper back skin temperature from the start of HT to the end of the 3rd trial was significantly lower in VEST than in CON (*p* < 0.05, end of HT, CON: 36.45 ± 1.1°C, VEST: 31.13 ± 1.93°C, CON: 36.8 ± 0.72°C, VEST: 32.36 ± 1.69°C). There were no significant differences between the two conditions in upper arm and thigh skin temperatures. There were no significant differences between the two conditions in deep thigh temperature (at the end of HT, CON: 37.2 ± 0.5°C, VEST: 37.5 ± 0.3°C).

### Heart Rate and Blood Lactate

**FIGURE 4:**
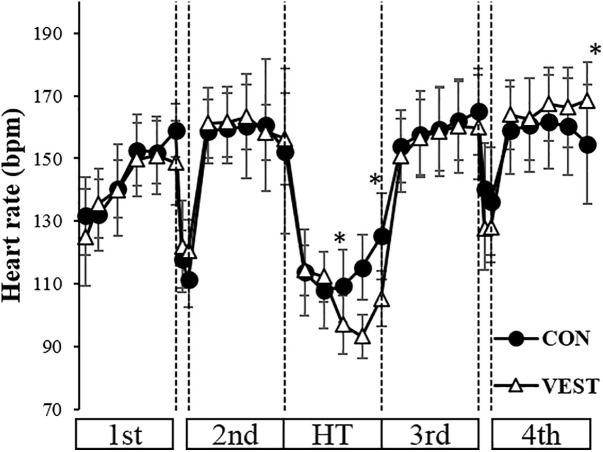
Change in HR during exercise protocol. ^∗^: significantly different between the two conditions. The values are shown as mean ± *SD. p* < 0.05.

Heart Rate was significantly lower in VEST during HT and higher in VEST at the last maximal pedaling than in CON (*p* < 0.05; Figure 4). Blood lactate at the end of warming up was 3.0 ± 1.2 mmol/L in CON and 3.3 ± 1.2 mmol/L in VEST, whereas at the end of the 4th trial, it was 5.3 ± 1.3 mmol/L in CON and 5.3 ± 1.8 mmol/L in VEST. Blood lactate did not differ between the two conditions.

### Body Fluid Balance

The rate of sweat loss was 1.8 ± 0.9% in CON and 1.5 ± 0.7% in VEST, and there were no significant differences between the two conditions. The urine specific gravity was 1.020 ± 0.006 in CON and 1.020 ± 0.007 in VEST before the exercise, whereas it was 1.023 ± 0.002 in CON and 1.023 ± 0.002 in VEST after the exercise. There were no significant differences in the urine specific gravity between the two conditions.

### Perceptual Index

**FIGURE 5:**
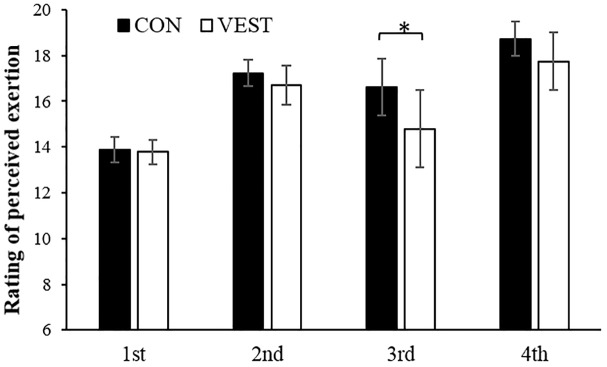
Change in RPE per trial. ^∗^: significantly different between the two conditions. The values are shown as mean ± *SD. p* < 0.05.

All perceptual data (TS, TC, and RPE) changed over the time during the intermittent exercise. TS was significantly lower in the 2nd half in VEST (Table 1). All TCs were significantly higher in the 2nd half in VEST (Table 1). Changes in RPE were not significantly different between the two conditions; however, the mean RPE per trial were not significantly lower in VEST (*p* < 0.05; Figure 5).

**Table 1:** Thermal sensation (TS) and thermal comfort (TC) during exercise protocol.

		Baseline	Post 1st	Post 2nd	Post HT	Post 3rd	Post 4th
*TS (whole)*	*CON*	2.3 ± 0.9	3.8 ± 0.6	5.8 ± 0.3	2.8 ± 1.0^∗^	5.1 ± 0.9^∗^	6 ± 0.0^∗^
	*VEST*	2.3 ± 1.1	3.6 ± 0.7	5.3 ± 0.5	-2.2 ± 1.4	3.8 ± 1.1	5.5 ± 0.5
*TS (neck)*	*CON*	2.5 ± 1.0	3.8 ± 0.6	5.8 ± 0.3	2.8 ± 1.0^∗^	5.1 ± 0.9^∗^	6 ± 0.0^∗^
	*VEST*	2.3 ± 1.1	3.6 ± 0.7	5.3 ± 0.5	-3.6 ± 0.1	3.8 ± 1.1	5.5 ± 0.5
*TS (upper)*	*CON*	2.3 ± 0.9	3.8 ± 0.6	5.8 ± 0.3	2.8 ± 1.0^∗^	5.1 ± 0.9^∗^	6 ± 0.0^∗^
	*VEST*	2.3 ± 0.9	3.6 ± 0.7	5.3 ± 0.5	-2.8 ± 0.8	3.8 ± 1.1	5.5 ± 0.5
*TC (whole)*	*CON*	-2.1 ± 0.6	-3.5 ± 0.5	-5.3 ± 0.9	-3.1 ± 1.2^∗^	-4.8 ± 0.9^∗^	-6 ± 0.0^∗^
	*VEST*	-2 ± 1.1	-3.5 ± 0.5	-5.1 ± 0.8	2.6 ± 1.8	-3.7 ± 1.1	-5.5 ± 0.5
*TC (neck)*	*CON*	-2.1 ± 0.6	-3.5 ± 0.5	-5.3 ± 0.9	-3 ± 0.8^∗^	-4.8 ± 0.9^∗^	-6 ± 0.0^∗^
	*VEST*	-2 ± 1.1	-3.5 ± 0.5	-5.1 ± 0.8	2.6 ± 1.8	-3.7 ± 1.1	-5.5 ± 0.5
*TC (upper)*	*CON*	-2.1 ± 0.6	-3.5 ± 0.5	-5.3 ± 0.9	-3.1 ± 0.9^∗^	-4.8 ± 0.9^∗^	-6 ± 0.0^∗^
	*VEST*	-1.9 ± 0.9	-3.5 ± 0.5	-5.1 ± 0.8	2.6 ± 1.8	-3.7 ± 1.1	-5.5 ± 0.5


*^∗^: significantly different between the two conditions. The values are shown as mean ± *SD. p* < 0.05.*

## Discussion

In this study, we investigated the effect of wearing a cooling vest, which cooled the torso and neck regions during HT, on intermittent exercise performance that imitated intermittent athletic games in the heat. We hypothesized that wearing a cooling vest decreases skin temperature and improves subjective sensation, and compared with no-cooling condition, subsequent intermittent exercise performance is improved in the heat. Our study suggests that wearing the cooling vest that covered the torso and neck during HT significantly improves intermittent exercise performance in the heat with decreased neck and mean skin temperature as well as improved subjective responses.

As reported in previous studies, wearing a cooling vest has a beneficial effect on skin temperatures, resulting in improved exercise performance. Therefore, in this study, it is considered that decreasing skin temperature by wearing the cooling vest improved performance. The mean skin temperature decreased by approximately 2.8°C through cooling, and it was significantly lower than of the CON until the end of the 3rd trial. Although ambient temperature or cooling time is different, the studies of Faulkner et al. (2015) and Schmit et al. (2017) indicated that the mean skin temperature decreased by approximately 3°C and 1.8°C, respectively, by wearing the cooling vest, and this reduction was maintained for approximately 15 min. Thus, this study showed that the mean skin temperature decreased as much as those in previous studies, which showed that wearing the cooling vest improves endurance exercise performance in the heat. Although the peripheral skin blood flow increases due to heat dissipation and the amount of blood supply to the skeletal muscle decreases (Bell et al., 1983; Gonzalez-Alonso et al., 1999), the reduction in skin temperature decreases the peripheral skin blood flow, and it potentially reduces cardiovascular strain and increases blood supply to the skeletal muscle (Sleivert et al., 2001; Gonzalez-Alonso and Calbet, 2003). Thus, in this study, wearing the cooling vest decreased skin temperature, which increases blood supply to the skeletal muscle; hence, participants were able to demonstrate higher power output even in the heat.

The decrease in neck skin temperature also positively influenced intermittent exercise performance. The neck skin temperature decreased by approximately 5°C by wearing the cooling vest, and it was significantly lower than that of the CON from HT to the end of the 3rd trial. The cooling vest used in this study was a new type of cooling vest, which can cool the neck, unlike the conventional one. Tyler et al. (2010) reported the improvement of endurance performance by cooling the neck during exercise without decreases in skin temperature, core temperature, and HR. In addition, it was reported that continuous neck cooling also has a beneficial effect on intermittent exercise performance (Sunderland et al., 2015). These improvements of performance are due to the fact that the neck is adjacent to the temperature-regulating centers and high sensory thermoreceptor (Shvartz, 1976; Gordon et al., 1990; Cotter and Taylor, 2005). Furthermore, it is possible that neck cooling decreased the cerebral temperature and affected mitigation of central fatigue. In the heat, it has been reported that central fatigue is caused by excessive increases in body temperature (Nybo and Nielsen, 2001), resulting in the decline of exercise performance (Hasegawa and Cheung, 2013). Ansley et al. (2008) improved the TS and submaximal exercise performance by cooling the face, and reported that cooling of the blood flow into the head decreases the cerebral temperature, which was considered as one of the factors that improve exercise performance. Furthermore, it is suggested that the neck cooling affects the arterial blood and a subsequent reduction in cerebral temperature (Caputa et al., 1986; Tyler and Sunderland, 2011). From the abovementioned studies, it is speculated that reduction of cerebral temperature and mitigation of central fatigue improve exercise performance, however, the current study did not measure cerebral temperature and cerebral blood flow. Thus, it is necessary to investigate these measurements in the future.

In this study, TS and TC were classified into parts and measured; all parts of TS and TC were significantly lower in VEST (*p* < 0.05). Participants felt the coolest at the end of HT in all parts of TS (respectively, whole: -2.2 ± 1.4, neck: -3.6 ± 1.0, upper: -2.8 ± 0.8), and participants felt that the neck was the coolest. Given that increases in skin temperature during exercise worsen thermal perception and TC (Flouris and Schlader, 2015), in the present study, it is considered that these improvements in thermal perception are due to the decrease in skin temperature (Schlader et al., 2011). The finding that the participants felt that the neck was the coolest, supports the assertion of a previous study reporting that thermoreceptive senses possibly differ depending on the body parts and region of the neck (Cotter and Taylor, 2005; Nakamura et al., 2013; Filingeri et al., 2014). However, in a previous study on neck cooling, TS of the neck significantly improved by neck cooling, but TS of the whole body was not significantly improved (Tyler and Sunderland, 2011). Thus, it was suggested that not only neck cooling but also upper body cooling, such as the chest or upper back, may be necessary for cooling the whole body and a cooling vest is useful for this. In addition, in the 3rd trial, the mean value of RPE was significantly lower in VEST. The difference in RPE is considered to be due to the improvement of TS and TC, and mean power outputs in the 3rd trial showed a high value despite the low RPE, indicating the usefulness of the cooling vest which covered the torso and neck. It has been suggested that sending thermal information to the hypothalamus indirectly affects reduction of power output (Tucker and Noakes, 2009). Therefore, mixed cooling of the torso and neck using the new type of cooling vest beneficially affects the subjective sensation, and the selective lower exercise intensity by the motor cortex was suppressed because of alleviation of thermal strain, which is sent from the peripheral thermoreceptors to the hypothalamus, thereby resulting in high performance as demonstrated by the participants.

In this study, intermittent exercise performance in the heat was improved without changes in Tre by improving the skin temperature, TS, and TC with the use of the cooling vest that cooled the neck, compared to that in CON. Tyler et al. (2010) reported that if an athlete is under sufficient thermal strain, time-trial performance in the heat can only be significantly enhanced by reducing neck temperature and TS, without significantly altering the physiological or peripheral neuroendocrinological responses to the exercise bout. However, improving performance due to reduction of skin temperature and TS without changes in Tre may be accompanied by a risk of heat illness. The neck region is an area of high allesthesia thermosensitivity and also is an area that can be cooled effectively (Tyler and Sunderland, 2011). Cooling the surface of the neck allowed the athletes to tolerate higher core temperature and HR (Tyler and Sunderland, 2011). However, it should be noted that the perceived level of thermal strain is dampened by cooling intervention, and the possibility of excessive core temperature increases due to high performance compared to no cooling. On the other hand, in this study, it was noteworthy that even though participants demonstrated high power output in VEST, Tre at the end of exercise was similar between VEST and CON. From these results, it was suggested that improving intermittent exercise performance by wearing the cooling vest does not cause excessive body temperature increases and does not increase the risk of athlete’s heat illness, and this method allowed athletes to play safely at the actual competition site.

Intermittent athletic games, such as soccer and rugby, have short rest periods between the exercise bouts; therefore, various countermeasures, such as convenient and practical cooling strategies, are considered to prevent the decline in exercise performance in the heat. In this study, the exercise protocol imitated intermittent exercise games, and wearing the cooling vest during HT improved intermittent exercise performance. In the cooling time of previous studies on cooling vests, Duffield and Marino (2007) applied the cooling vest for 15 min before exercise and 10 min during HT, whereas Castle et al. (2006) applied the cooling vest for 20 min before exercise. Compared to these previous studies, sprint performance improved in this study despite the short cooling time, because the cooling vest was able to cool the neck. Furthermore, it was important to note that the degree of lowering deep thigh temperature of the working muscle was similar between VEST and CON, suggesting that the cooling vest had no negative effects on subsequent intermittent exercise. The previous study reported that application of ice packs on the thigh for 45 min had a negative effect on subsequent full-pedaling performance (Sleivert et al., 2001). Thus, it is suggested that direct cooling on the working muscle may influence the subsequent high-intensity exercise performance and sufficient re-warming is required. That is, direct cooling on the working muscle in a short time, such as HT, may not be suitable, and this study showed that the cooling vest is the appropriate cooling strategy during short resting periods. Therefore, the application of the new type of cooling vest, which cooled the neck in a relatively short time, improved subsequent intermittent exercise performance without lowering the performance of working muscle. This is considered to be the attractive cooling strategy for athletes involved in intermittent sporting activities.

In previous studies on cooling vests, wearing the cooling vest improved exercise performance without improvement of physiological index, such as core temperature or HR (Faulkner et al., 2015; Schmit et al., 2017). In contrast, in this study, HR was significantly lower in VEST during HT and higher in VEST at the last maximal pedaling. The reason for this improvement is that the cooling vest used in this study was able to cool the neck. By wearing the cooling vest, the peripheral region, such as the skin, is cooled down and the necessity of heat dissipation is alleviated. As a result, it can be predicted that the HR decreased because the blood flow in the center increased and the cardiovascular strain was alleviated. However, in the previous study showing improved intermittent performance by cooling the neck, HR was not significantly decreased only by neck cooling (Tyler and Sunderland, 2011). Therefore, it is considered that mixed cooling of the torso and neck is necessary for the improvement of physiological indexes other than skin temperature; hence, our hypothesis that cooling vests covers torso and neck are useful for intermittent exercise performance.

## Limitation

The limitation of this study was the reproducibility of intermittent athletic game. First, participants spent HT in the heat, but, in an actual intermittent game, the player spends the rest period in the locker room in which room temperature is cooled by air conditioning. In this study, we wanted to investigate the effects of the cooling vest itself, thus participants spent HT in the heat. However, in the future, it is necessary to set a more practical rest period. Second, there was an issue associated with exercise intensity. Özgünen et al. (2010) investigated the influence of the heat on physiological index and performance during actual soccer games. They reported that the peak core temperature was observed at the end of the 1st half. However, in this study, the peak core temperature was observed later in the 2nd half, which differed from the result of the actual competition. These varying results are considered to be caused by the configuration of the exercise intensity. In this protocol, since the exercise intensity was controlled except for the 5-s maximal pedaling, the exercise task of all active recovery had the same exercise intensity. However, in the actual competition, a decline in performance was also observed in other parameters such as jogging in the 2nd half compared to the 1st half (Özgünen et al., 2010). Therefore, in this study, it is considered that the active recovery had the same intensity in the 1st half and the 2nd half, and it resulted in the same value of Tre between the two conditions. In future research, exercise intensity other than maximal pedaling should not be controlled, and evaluation of exercise performance will also be necessary. In addition, we could not measure skin blood flow, cerebral temperature, muscle blood flow, and blood pressure in this study. In future research, to clarify the mechanism of circulatory dynamics, it is necessary to measure these indexes.

## Conclusion

By wearing the cooling vest which cools the neck and upper torso regions during HT that imitates intermittent athletic competitions, such as soccer, in the heat, physiological indicators, such as skin temperature and HR, and subjective sensation, such as TS or TC, were significantly improved, and subsequent intermittent exercise performance improved. In addition, as the improvement in performance was observed in a relatively short cooling time, the cooling vest is more practical to use than the cooling interventions requiring large-scale facilities such as a cold water immersion, suggesting that wearing the cooling vest is effective at the actual competition site.

## Author Contributions

YC designed the study with assistance from HH. All authors completed the data collection, data analysis, and manuscript preparation.

## Conflict of Interest Statement

The authors declare that the research was conducted in the absence of any commercial or financial relationships that could be construed as a potential conflict of interest.
